# High-fidelity initialization and control of electron and nuclear spins in a four-qubit register

**DOI:** 10.1038/s41565-023-01596-9

**Published:** 2024-02-07

**Authors:** J. Reiner, Y. Chung, S. H. Misha, C. Lehner, C. Moehle, D. Poulos, S. Monir, K. J. Charde, P. Macha, L. Kranz, I. Thorvaldson, B. Thorgrimsson, D. Keith, Y. L. Hsueh, R. Rahman, S. K. Gorman, J. G. Keizer, M. Y. Simmons

**Affiliations:** 1 Centre of Excellence for Quantum Computation and Communication Technology, School of Physics, University of New South Wales, Sydney, New South Wales Australia; 2 Silicon Quantum Computing Pty Ltd., University of New South Wales, Sydney, New South Wales Australia; 3 School of Physics, University of New South Wales, Sydney, New South Wales Australia

**Keywords:** Electronic devices, Quantum information, Nanoscale devices, Qubits

## Abstract

Single electron spins bound to multi-phosphorus nuclear spin registers in silicon have demonstrated fast (0.8 ns) two-qubit $\sqrt{\mathrm{SWAP}}$ gates and long spin relaxation times (~30 s). In these spin registers, when the donors are ionized, the nuclear spins remain weakly coupled to their environment, allowing exceptionally long coherence times. When the electron is present, the hyperfine interaction allows coupling of the spin and charge degrees of freedom for fast qubit operation and control. Here we demonstrate the use of the hyperfine interaction to enact electric dipole spin resonance to realize high-fidelity ($F=10{0}_{-6}^{+0}$%) initialization of all the nuclear spins within a four-qubit nuclear spin register. By controllably initializing the nuclear spins to $\left\vert \Downarrow \Downarrow \Downarrow \right\rangle$, we achieve single-electron qubit gate fidelities of *F* = 99.78 ± 0.07% (Clifford gate fidelities of 99.58 ± 0.14%), above the fault-tolerant threshold for the surface code with a coherence time of ${T}_{2}^{\,* }=12\,\upmu {{{\rm{s}}}}$.

## Abstract (web summary)

An electric dipole spin resonance protocol making use of hyperfine interaction enacts high-fidelity initialization of a four-qubit nuclear spin register in silicon. This protocol allows for high-fidelity qubit control and a path towards a register-based quantum computer using the exceptional coherence properties of donors in silicon.

## Main

Quantum bits encoded into the spin of individual electrons in silicon have shown rapid progress in the past few years^1^. Phosphorus atom qubits in silicon (Si:P), in particular, have allowed for single qubit gates using both electron spin resonance (ESR)^2^ and nuclear spin resonance (NMR)^3^ with some of the highest fidelities and longest coherence times in the solid state to date^4–8^ (Table 1). One of the unique control mechanisms in these atom-based systems is the hyperfine coupling between the electron and nuclear spins, which has been utilized to demonstrate violation of Bell inequalities^6^ and a high-fidelity two-qubit gate^8^. As we advance to many qubits in these multi-nuclear spin registers, the hyperfine coupling has also be used for individual qubit addressability in silicon^8–10^ and diamond^11–14^.

**Table 1:** Comparison of nuclear spin registers realized by phosphorus atom qubits in silicon (Si:P) and NV colour centres in diamond (C:NV)

Parameter	Si:P	C:NV
Fabrication accuracy	±0.384 nm (ref. ^43^)	±5 nm (ref. ^26^)
Dopant activation	100% (ref. ^43^)	2.5% (ref. ^44^)
Qubit readout fidelity	99.95% (ref. ^35^)	95% (ref. ^12^)
Qubit readout speed	1.5 μs (ref. ^7^)	10 μs (ref. ^12^)
Electron spin fidelity	99.93% (ref. ^5^)	99.99% (ref. ^14^)
Electron spin gate speed	2.3 μs (ref. ^5^)	1 μs (ref. ^14^)
Electron–photon gate	—	70% (ref. ^45^)
Electron–electron gate	87% (ref. ^28^)	—
Nuclear spin fidelity	99.98% (ref. ^4^)	99.1% (ref. ^13^)
Nuclear spin gate speed	19 μs (ref. ^4^)	400 μs (ref. ^13^)
Nuclear–nuclear gate	99.37% (ref. ^8^)	95% (ref. ^13^)

In this Article, we address the importance of the hyperfine interaction for a different role: the initialization and control of electron and nuclear spins in these multi-qubit registers critical for the development of a large-scale quantum computer^15,16^. The initialization of nuclear spins in nitrogen vacancy (NV) centres in diamond has to date used either non-deterministic methods such as post-selection or more active methods such as NMR-based initialization to perform electron–nuclear SWAP operations to map the electron spin state to the individual nuclear spin^13,17–19^. Similarly, initial experiments in Si:P multi-nuclear spin registers have relied on post-selection to initialize the nuclear spin qubits^8^. While these non-deterministic methods work well for small qubit systems, when scaling to a large quantum computer with multiple coupled registers, the time required to initialize the system will become unworkable. Here, we propose a robust method for initialization of nuclear spins in these multi-qubit registers using the hyperfine interaction with direct SWAP operations to initialize and control both electron and nuclear spins in a four-qubit register.

Phosphorus donor atom qubits represent an ideal system to investigate nanoscale multi-nuclear spin registers, since sub-nanometre precision placement of the donors has already been realized^20–24^, an on-going challenge for the diamond system^25–27^. Such precision not only allows for direct inter-register coupling—a highly desirable property for scalability—but has demonstrated other benefits, including long (~30 s) spin relaxation times^21^, highly tunable exchange coupling^22^, fast electron–electron two-qubit gate operations^28^ between registers and enhanced qubit addressability while minimising errors on neighbouring spectator qubits^10,20^. Using the electron exchange interactions, we can envisage a large-scale modular architecture based on coupled multi-nuclear spin registers^15^. Phosphorus donors in silicon also show 100% dopant activation compared with the few percent of NV centres. While the rate of NV formation being in the negatively charged state is quite low after nitrogen implantation, the position of the active NV centre can be selected by the measurement.

### Multi-nuclear spin qubit registers in silicon

**Fig. 1:**
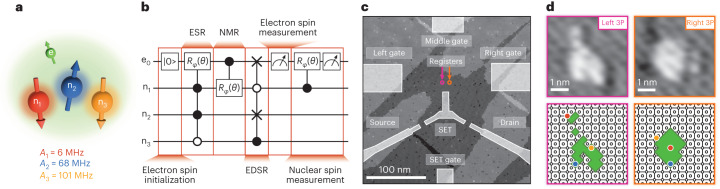
Four-qubit spin registers in isotopically purified silicon-28. **a**, A schematic of a multi-donor spin register in silicon. The four-qubit register comprises an electron spin qubit directly coupled to three phosphorus nuclear spin qubits via the hyperfine interaction. **b**, Multi-qubit operations in a four-qubit register include electron spin initialization via energy-selective readout, ESR for controlled-rotation gates (*R*_*ϕ*_(*θ*)) of the electron conditional on the nuclear spin state, NMR for controlled-rotation gates (*R*_*ϕ*_(*θ*)) of the nuclei conditional on the electron spin state, EDSR, electron spin measurement and ancilla-based nuclear spin measurement. **c**, STM image of two individual four-qubit registers (dots) used to perform EDSR for nuclear spin initialization. **d**, Lithographic patches used to define the left and right multi-nuclear spin registers, both containing three phosphorus atoms. The predicted atomistic locations of these donors (red, yellow, and blue) within each lithographic patch (green region) are based on the measured hyperfine and relaxation times compared with atomistic tight-binding simulations (Supplementary Information I).

Here, we demonstrate a deterministic protocol for initializing all the nuclear spins in a four-qubit donor nuclear spin register (Fig. 1a) using the hyperfine interaction to mediate electric dipole spin resonance (EDSR). To date, the EDSR mechanism has been proposed for qubit control^16,29^ and long-range spin coupling^30^ in flip–flop qubit architectures, where the two basis states are the ($\left\vert \Downarrow \uparrow \right\rangle$) and ($\left\vert \Uparrow \downarrow \right\rangle$) states of a donor delocalized across two different orbital states. Now, we show that it can also be operated within a single multi-donor qubit register utilizing the hyperfine interaction. Importantly, we show how this hyperfine-mediated EDSR mechanism can enact unique controlled-multi-qubit SWAP gates (the Fredkin gate^31^) that, when combined with electron spin state preparation, transfer the polarization of the electron onto the nuclear spins^17,32,33^. This protocol can be repeated multiple times to achieve robust nuclear spin initialization fidelity above 99%. This new EDSR initialization protocol offers an attractive alternative to non-deterministic measurement-based methods that require additional overheads in time due to their probabilistic nature. NMR-based initialization methods are also slower owing to the weak coupling of the magnetic field to the nuclear spin and require vastly different frequency ranges and multiple ESR/NMR pulses for high-fidelity operation. The use of similar qubit frequencies in this new protocol therefore greatly reduces the design complexity of control structures and allows for narrow-band operation of the qubits.

To investigate the operation of our multi-nuclear spin qubit registers, we precision-pattern multi-donor quantum dots using scanning tunnelling microscopy (STM) hydrogen lithography^10^ (Supplementary Information I). Figure 1c shows an STM image of the patterned device, where the bright regions correspond to regions in which the hydrogen mask has been selectively removed to allow incorporation of phosphorus donors via phosphine dosing. The device consists of two small quantum dot registers, each defined by three phosphorus atoms (3P) (Fig. 1d and Supplementary Information I) and tuned to contain one electron spin each. These quantum dots are weakly tunnel-coupled to a single-electron transistor patterned ~17 nm away, which acts as both a charge sensor and an electron reservoir to load the electrons onto the nuclear spin registers^34^. High-frequency electromagnetic fields for ESR and EDSR control are generated using an on-chip microfabricated antenna on the surface^10^. The electron spin qubits are tuned using electrostatic gates labelled Left gate, Middle gate, and Right gate, and the single-electron transistor (SET) conductance can be tuned using the SET gate. Electron spin initialization and readout is performed using a ramped spin measurement^35^, giving a fidelity of ~81% at an electron temperature of ~200 mK at magnetic field *B* = 1.45 T.

**Fig. 2:**
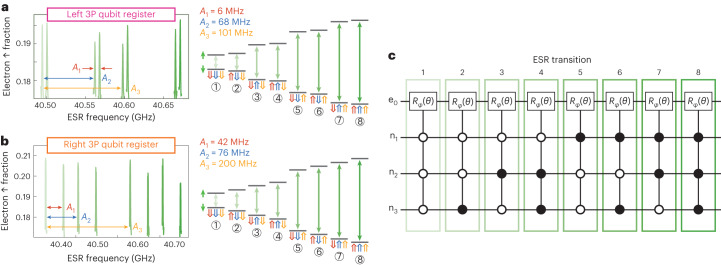
ESR for the left and right four-qubit registers. **a**,**b**, Left: The ESR spectrum for an electron on the left register (**a**) and for the right register (**b**) at a magnetic field of *B* = 1.45 T. Right: The corresponding schematic energy level diagrams for the 3P spin registers with a single electron spin (*↑* or *↓* depending on the different nuclear spin ⇑ or ⇓ states). Each ESR transition is shown by the green arrows. **c**, The circuit representation of the different ESR transitions in the four-qubit nuclear spin registers. Full circles indicate conditional on $\left\vert \Uparrow \right\rangle$. Empty circles indicate conditional on $\left\vert \Downarrow \right\rangle$.

The spin Hamiltonian for an individual *N*-donor quantum dot (multi-nuclear spin register) is given by (with *ℏ* = 1)

1 $$H={\gamma }_{\mathrm{e}}{{{\bf{B}}}}\cdot {{{\bf{S}}}}+\mathop{\sum }\limits_{i}^{N}{\gamma }_{\mathrm{n}}{{{\bf{B}}}}\cdot {{{{\bf{I}}}}}_{i}+\mathop{\sum }\limits_{i}^{N}{{{\bf{S}}}}\cdot \bar{{A}_{i}}({{{\bf{E}}}})\cdot {{{{\bf{I}}}}}_{i},$$

where **B** is the external magnetic field, *γ*_e_ (*γ*_n_) is the electron (nuclear) gyromagnetic ratio, **S**(**I**_*i*_) is the vector of Pauli spin operators for the electron (*i*th nuclei) and $\bar{{A}_{i}}({{{\bf{E}}}})$ is the electric field-dependent hyperfine tensor. The hyperfine interaction can be considered as an effective magnetic field that the *i*th nuclear spin and the electron spin impart on each other. The presence of the hyperfine interaction therefore lifts the degeneracy of the nuclear spin frequencies and splits the ESR frequencies into nuclear spin-dependent transitions. We observe 2^3^ = 8 distinct peaks in the electron spin-up probability (Fig. 2a,b, left), corresponding to flipping the electron spin conditional on the state of all three nuclear spins^10^, as illustrated in the energy diagrams to the right. In these measurements, all eight ESR transitions of both registers are observed since all the nuclear spin states are populated over the course of the experiment. Here, the total measurement time (on the order of minutes) is longer than the spontaneous nuclear spin flip times (order of seconds) that can be caused by ionization shock^3,10^ and hyperfine-mediated relaxation^36^. Since each of these electron spin transitions is dependent on the state of the individual nuclear spins, we can perform controlled-rotation gates conditional on all the nuclear spin states by driving one or multiple electron transitions (Fig. 2c). These multi-qubit gates arise from the contact hyperfine interaction of the electron spin with the nuclear spins, resulting in nuclear spin-dependent transitions of the electron spin. From the measured hyperfine values *A*_1_, *A*_2_, and *A*_3_ obtained from the ESR spectra as well as the electron and nuclear gyromagnetic ratios, we can directly calculate the resonance frequencies of all the relevant EDSR transitions by finding the spin-conserving transitions in equation (1) (Supplementary Information II).

### EDSR for initialization of the qubit register

**Fig. 3:**
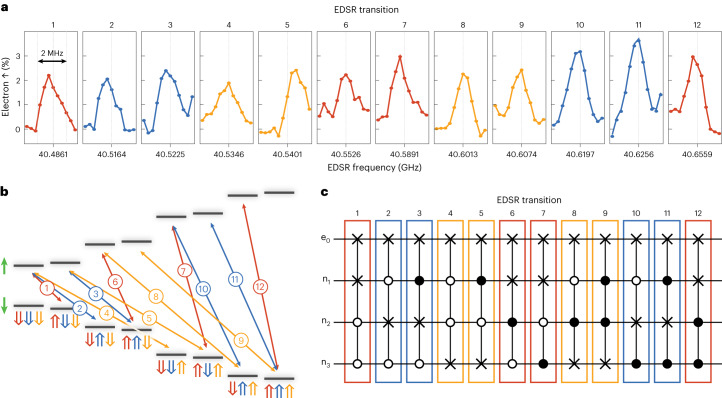
EDSR of the left four-qubit register. **a**, The measured EDSR spectrum of the left register, showing 12 resonances corresponding to angular momentum-conserving electron–nuclear flip flops measured through the electron spin state. The different colours indicate which phosphorus nuclear spin is flipped (red, n_1_; blue, n_2_; orange, n_3_). **b**, The corresponding energy level diagram for the four-qubit register, showing the 12 EDSR transitions measured in **a**. **c**, Equivalent gate operations corresponding to each of the 12 EDSR transitions. The controlled-SWAP (Fredkin gates) for each nuclear spin configuration (black controls indicate conditional on $\left\vert \Uparrow \right\rangle$ and white controls indicate conditional on $\left\vert \Downarrow \right\rangle$) can be used for robust initialization of the nuclear spins in the four-qubit register.

Modulation of the electric field around the spin register creates a time-dependent shift of the hyperfine interaction, which when applied on-resonance with the EDSR transition frequencies, drives electron–nuclear flip flops^16^. Figure 3a shows the EDSR spectrum for the left spin register measured via readout of the electron spin with *N*2^*N*−1^ = 12 (where *N* = 3 is the number of P atoms) transitions since for each nuclear spin there are 2^*N*−1^ transitions due to the states of the other nuclei (Fig. 3b). For the remainder of the paper, we focus solely on the left spin register. Importantly, the EDSR frequencies correspond to the 12 different Fredkin gates (controlled-SWAP gates) possible between the electron spin and each of the nuclear spins (Fig. 3c). Four of these 12 transitions correspond to the flip of nuclear spin n_1_ (red) where the other two nuclear spins remain $\left\vert \Downarrow \Downarrow \right\rangle$, $\left\vert \Downarrow \Uparrow \right\rangle$, $\left\vert \Uparrow \Downarrow \right\rangle$ and $\left\vert \Uparrow \Uparrow \right\rangle$. Four correspond to the flip of nuclear spin n_2_ (blue), and the final four to nuclear spin n_3_ (orange). As an example, transition 1 corresponds to a controlled-controlled-SWAP between the electron spin and the third nuclear spin, $\left\vert \downarrow \Downarrow \Downarrow \Uparrow \right\rangle \to \left\vert \uparrow \Downarrow \Downarrow \Downarrow \right\rangle$. To be able to observe all possible EDSR transitions, we introduce a pulse sequence that first randomizes the nuclear configuration and then initializes the electron spin to $\left\vert \downarrow \right\rangle$ before applying an adiabatic inversion pulse at the expected EDSR frequencies to flip the electron to the $\left\vert \uparrow \right\rangle$ manifold, followed by readout of the electron spin. For efficient inversion of the electron spin, we sweep a 3 MHz window around each EDSR frequency (Supplementary Information II) with the resulting spectra shown in Fig. 3a. We find excellent agreement between the experimental and theoretical EDSR frequencies to within ~1 MHz.

**Fig. 4:**
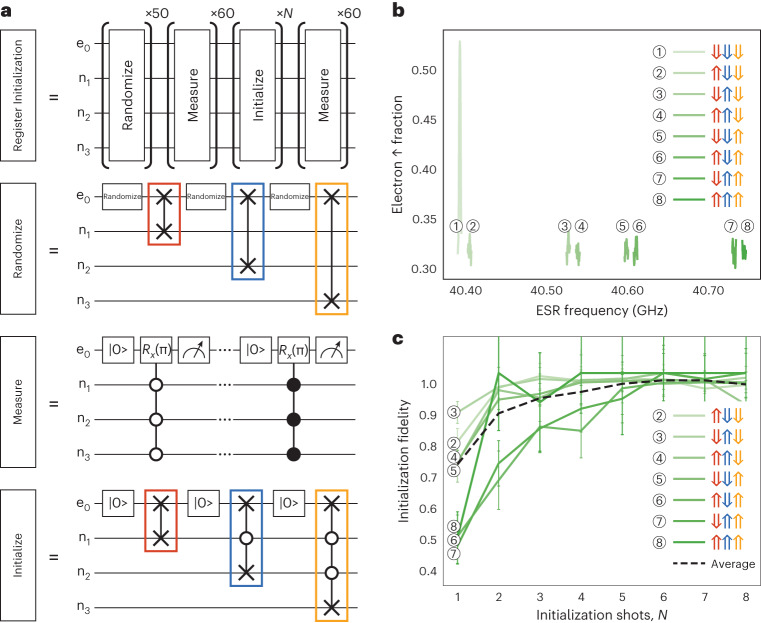
High-fidelity nuclear spin initialisation in a four-qubit register. **a**, The sequence for the estimation of the initialization fidelity of the qubit register with four sequential gate sequences of native multi-qubit gates between the electron and nuclear spin qubits: randomize, measure, initialize and measure. Randomization of the nuclear spin register is performed by initializing the electron spin into a mixed state and performing SWAP gates between the electron and nuclear spins via adiabatic EDSR. Measurement involves controlled-π rotations of the electron spin conditional on the total nuclear spin state, followed by a measurement of the electron spin. The initializtion step involves initialization of the electron spin followed by SWAP gates between the electron and each of the nuclear spins. **b**, The resulting ESR spectrum after initialization of the qubit register shows that the nuclear spins are polarized into the $\left\vert \Downarrow \Downarrow \Downarrow \right\rangle$ state (at the lowest ESR transition). **c**, The initialization fidelity of the nuclear spins into $\left\vert \Downarrow \Downarrow \Downarrow \right\rangle$ as a function of the number of repetitions (*N*) of the initialization protocol for different initial nuclear spin states. Error bars correspond to ±1 s.d. about the mean. The fidelity increases for more iterations, indicating robustness. That is, the initialization protocol can be applied multiple times to increase the fidelity to achieve $F=10{0}_{-6}^{+0}$%.

Having identified the different EDSR transitions in the left 3P qubit register, we now investigate the use of controlled-SWAP gates between the electron and nuclear spins to initialize the nuclear spins by polarization transfer from the electron spin qubit. The overall nuclear spin initialization fidelity depends strongly on the efficiency of each EDSR inversion pulse, which requires a careful optimization between the EDSR power and the pulse duration using Landau–Zener interferometry (Supplementary Information III). Figure 4a shows the sequence used to determine the initialization fidelity, in this case into the $\left\vert \Downarrow \Downarrow \Downarrow \right\rangle$ state. Characterization of the initialization fidelity requires four steps: randomize, measure, initialize and measure (see Supplementary Information IV for details on the randomize and measure steps). Each of these four individual operations involves the use of multi-qubit gates between the electron and the nuclear spins in the register.

The initialize step is as follows: Once the nuclear spin state has been randomized and measured, we then perform our nuclear spin initialization procedure to initialize the $\left\vert \Downarrow \Downarrow \Downarrow \right\rangle$ state. The initialization procedure works by swapping the electron $\left\vert \downarrow \right\rangle$ state with each of the nuclear spin states. To initialize to $\left\vert \Downarrow \Downarrow \Downarrow \right\rangle$, starting from a random nuclear configuration, we first initialize the electron spin to the $\left\vert \downarrow \right\rangle$ state and apply four EDSR pulses (1, 6, 7 and 12) sequentially (Fig. 3c), which leaves n_1_ in the $\left\vert \Downarrow \right\rangle$ state. This initialization sequence transfers the spin-down polarization from the electron to nuclear spin n_1_, unconditional on the states of the other two nuclear spins. We then repeat the electron spin initialization and apply EDSR pulses 2 and 10 only to initialize spin n_2_. This is conditional on spin n_1_ already being $\left\vert \Downarrow \right\rangle$, since we have just initialized that nuclear spin. To conclude the nuclear initialization sequence, we then initialize the electron spin down again and apply EDSR 4, realizing a single controlled-SWAP gate between the electron spin and spin n_3_ conditional on spins n_1_ and n_2_ being $\left\vert \Downarrow \right\rangle$. Therefore, the total initialization sequence is {$\left\vert \downarrow \right\rangle$, 1, 6, 7, 12, $\left\vert \downarrow \right\rangle$, 2, 10, $\left\vert \downarrow \right\rangle$, 4}. Importantly, we can initialize into any arbitrary nuclear spin state by modifying this pulse sequence and/or by initializing the electron $\left\vert \uparrow \right\rangle$ before swapping with the nuclear spin (Supplementary Information VI). Finally, to determine the nuclear spin state after the initialization protocol, we perform an ESR measurement. In Fig. 4b, we see that the electron spin is only inverted when driving the lowest ESR peak, corresponding to the polarized nuclear spin state $\left\vert \Downarrow \Downarrow \Downarrow \right\rangle$.

From measuring the nuclear configuration before and after this initialization sequence, we use a Markov model to construct a normalized transition probability matrix *M*. The initialization fidelity from the initial state *i* to the final nuclear spin state *j* is then given by the *M*_*j*,*i*_ element (Supplementary Information VI). Figure 4c shows the initialization fidelity achieved starting from each one of the seven possible nuclear spin states to the target state $j=\left\vert \Downarrow \Downarrow \Downarrow \right\rangle$ (excluding *i* = *j* when the initial state is already at the target state) as a function of the number of initialization attempts *N*. These curves are normalized by the probability of starting and remaining in the target state (*i* = *j* case), which helps to remove the nuclear state readout and electron initialization errors and leads to uncertainty in the total probability (Fig. 4c). Owing to the low Stark coefficient of n_3_, the EDSR Rabi frequency for these transitions is the smallest and hence the inversion fidelity is lower compared with n_1_ and n_2_ (Supplementary Information III). As a consequence, we see in Fig. 4c that the fidelity for n_3_ to flip is much lower for *N* < 5. However, by averaging over the different initial nuclear spin configurations (dashed black line), we obtain an overall effective initialization fidelity of $\bar{F}=10{0}_{-6}^{+0} \%$ for *N* ≥ 5 repetitions.

### High-fidelity control of the electron spin qubit

**Fig. 5:**
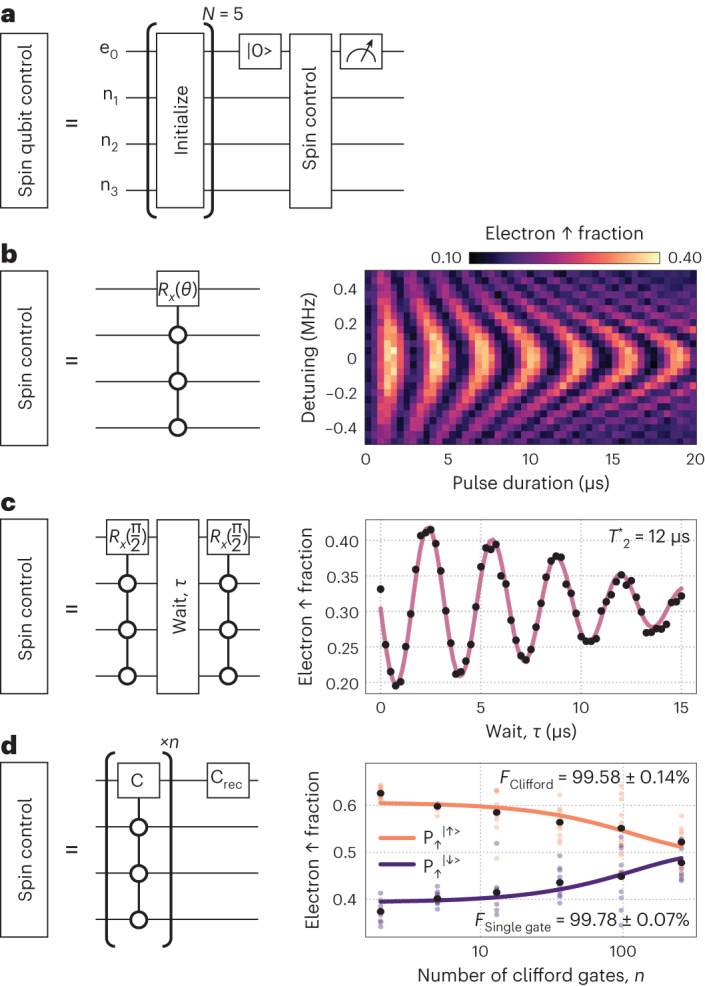
Single-electron spin qubit operation in a four-qubit nuclear spin register. **a**, Circuit diagram of the initialization of the nuclear spin qubits followed by operation of the electron spin qubit for three different experiments. **b**, Circuit diagram and Rabi oscillations used to create a Rabi–Chevron pattern by varying the ESR frequency and pulse time, demonstrating coherent control of the bound electron spin. **c**, Circuit diagram and Ramsey decay measurement of the electron spin qubit with ${T}_{2}^{\,* }=12\,\upmu {{{\rm{s}}}}$. **d**, Circuit diagram and randomized benchmarking of the electron spin qubit corresponding to an average Clifford gate fidelity of *F*_Clifford_ = 99.58 ± 0.14% and primitive gate fidelity of *F*_Single gate_ = 99.78 ± 0.07%. Here, we define P_↑_^|↑ >^ to be the measured probability, P_↑_, when the target state was |↑>; C is Clifford gate; C_rec_ is the recovery Clifford gate.

Having achieved $F=10{0}_{-6}^{+0} \%$ nuclear spin initialization fidelity, we now initialize the register to $\left\vert \Downarrow \Downarrow \Downarrow \right\rangle$ to demonstrate high-fidelity control of the single electron spin qubit in this multi-nuclear spin qubit register. Importantly, this can now be achieved at a single resonance frequency (that is, ESR conditional on the nuclear spins being in the $\left\vert \Downarrow \Downarrow \Downarrow \right\rangle$ state) and does not require any post-selection (Fig. 5a, circuit diagram). Figure 5b shows a Rabi–Chevron (electron spin rotations as a function of frequency and pulse duration) measurement on the electron spin qubit when initialized to $\left\vert \Downarrow \Downarrow \Downarrow \right\rangle$ of the left spin register where we measure ${T}_{2}^{\mathrm{Rabi}}=180\,\upmu {{{\rm{s}}}}$ with a gate time of *t*_π_ = 1.64 μs. We have also measured the dephasing time, ${T}_{2}^{\,* }=12\,\upmu {{{\rm{s}}}}$ with a measurement time of 300 s through the Ramsey sequence shown in Fig. 5c, comparable to previous donor-based^8,37^ and gate-defined quantum dot single spin qubits^38,39^ (${T}_{2}^{\,* } \approx 10\,\upmu {{{\rm{s}}}}$). This value of ${T}_{2}^{\,* }$ validates the advantage of using solid source isotopically purified silicon-28 for molecular beam epitaxial growth, which has now been incorporated into our atomic-precision manufacturing process.

Finally, we performed randomized benchmarking on the electron spin qubit to determine the Clifford gate fidelity (Fig. 5d). Here, the nuclear spins are initialized into $\left\vert \Downarrow \Downarrow \Downarrow \right\rangle$ using five initialization shots and the electron spin is controlled via coherent ESR drive conditional on the nuclear spins being in $\left\vert \Downarrow \Downarrow \Downarrow \right\rangle$ achieved via initialization. We obtain an average Clifford gate fidelity of  *F*_Clifford_ = 99.58 ± 0.14%, corresponding to a primitive gate fidelity of *F*_Single gate_ = 99.78 ± 0.07%, above the 99% minimum fidelity required for fault-tolerant surface code error correction (Supplementary Information VI). This primitive gate fidelity is comparable to other semiconductor spin qubits^4,38,40^ fabricated in isotopically purified silicon. Importantly, we can now access multi-nuclear spin registers using our precision manufacturing route to achieving high-fidelity spin qubits.

## Conclusions

We demonstrate high-fidelity initialization and control of electron and nuclear spins in a four-qubit register defined using phosphorus donors in silicon. By initializing the nuclear spins to $\left\vert \Downarrow \Downarrow \Downarrow \right\rangle$ with a fidelity $F=10{0}_{-6}^{+0} \%$, we demonstrated coherent control of the electron spin qubit with ${T}_{2}^{\,* }=12\,\upmu {{{\rm{s}}}}$ and gate fidelities *F*_G_ = 99.78 ± 0.07% using randomized benchmarking. This unique control of both electron and nuclear spins within this multi-qubit register allows for the future development of small-scale algorithms. Immediate future work will focus on improving the per-shot EDSR initialization fidelity, using optimized EDSR inversion efficiencies by engineering larger electric fields in the device and/or performing higher-fidelity electron spin initialization by decreasing the electron temperature. We also aim to incorporate coherent resonant EDSR control of the nuclear spins. This can be achieved by optimizing the electric field coupling using larger Stark coefficients and/or increasing the electric field strength by incorporating electrical control via in-plane phosphorus-doped silicon gates rather than through the broadband antenna line. By coherently controlling the nuclear spin qubits in the register, we can access a rich four-qubit system with multiple controlled (SWAP and CNOT) gates for efficient algorithms in the noisy intermediate-scale quantum era^41,42^.

## Online content

Any methods, additional references, Nature Portfolio reporting summaries, source data, extended data, supplementary information, acknowledgements, peer review information; details of author contributions and competing interests; and statements of data and code availability are available at 10.1038/s41565-023-01596-9.

### Supplementary information

Supplementary Information Supplementary discussion with six sections, four figures and three tables.

## Data Availability

The data pertaining to this study are available from the corresponding author upon reasonable request.

## References

[1] Burkard G, Ladd TD, Pan A, Nichol JM, Petta JR (2023). Semiconductor spin qubits. Rev. Mod. Phys..

[2] Pla JJ (2012). A single-atom electron spin qubit in silicon. Nature.

[3] Pla JJ (2013). High-fidelity readout and control of a nuclear spin qubit in silicon. Nature.

[4] Muhonen JT (2014). Storing quantum information for 30 seconds in a nanoelectronic device. Nat. Nanotechnol..

[5] Muhonen J (2015). Quantifying the quantum gate fidelity of single-atom spin qubits in silicon by randomized benchmarking. J. Phys. Condens. Matter.

[6] Dehollain JP (2016). Bell’s inequality violation with spins in silicon. Nat. Nanotechnol..

[7] Keith D (2019). Single-shot spin readout in semiconductors near the shot-noise sensitivity limit. Phys. Rev. X.

[8] Mądzik MT (2022). Precision tomography of a three-qubit donor quantum processor in silicon. Nature.

[9] Fricke L (2021). Coherent control of a donor-molecule electron spin qubit in silicon. Nat. Commun..

[10] Hile SJ (2018). Addressable electron spin resonance using donors and donor molecules in silicon. Sci. Adv..

[11] Doherty MW (2013). The nitrogen-vacancy colour centre in diamond. Phys. Rep..

[12] Abobeih MH (2018). One-second coherence for a single electron spin coupled to a multi-qubit nuclear-spin environment. Nat. Commun..

[13] Bradley CE (2019). A ten-qubit solid-state spin register with quantum memory up to one minute. Phys. Rev. X.

[14] Rong X (2015). Experimental fault-tolerant universal quantum gates with solid-state spins under ambient conditions. Nat. Commun..

[15] Hill CD (2015). Quantum computing: a surface code quantum computer in silicon. Sci. Adv..

[16] Tosi G (2017). Silicon quantum processor with robust long-distance qubit couplings. Nat. Commun..

[17] Gurudev Dutt MV (2007). Quantum register based on individual electronic and nuclear spin qubits in diamond. Science.

[18] Robledo L (2011). High-fidelity projective read-out of a solid-state spin quantum register. Nature.

[19] Taminiau TH, Cramer J, Sar TVD, Dobrovitski VV, Hanson R (2014). Universal control and error correction in multi-qubit spin registers in diamond. Nat. Nanotechnol..

[20] Büch H, Mahapatra S, Rahman R, Morello A, Simmons MY (2013). Spin readout and addressability of phosphorus-donor clusters in silicon. Nat. Commun..

[21] Watson TF (2017). Atomically engineered electron spin lifetimes of 30 s in silicon. Sci. Adv..

[22] Broome MA (2018). Two-electron spin correlations in precision placed donors in silicon. Nat. Commun..

[23] Kranz L (2022). The use of exchange coupled atom qubits as atomic-scale magnetic field sensors. Adv. Mater..

[24] Kiczynski M (2022). Engineering topological states in atom-based semiconductor quantum dots. Nature.

[25] Waldermann F (2007). Creating diamond color centers for quantum optical applications. Diamond Relat. Mater..

[26] Pezzagna S, Naydenov B, Jelezko F, Wrachtrup J, Meijer J (2010). Creation efficiency of nitrogen-vacancy centres in diamond. N. J. Phys..

[27] Deák P, Aradi B, Kaviani M, Frauenheim T, Gali A (2014). Formation of NV centers in diamond: a theoretical study based on calculated transitions and migration of nitrogen and vacancy related defects. Phys. Rev. B.

[28] He Y (2019). A two-qubit gate between phosphorus donor electrons in silicon. Nature.

[29] Krauth F (2022). Flopping-mode electric dipole spin resonance in phosphorus donor qubits in silicon. Phys. Rev. Appl..

[30] Osika EN (2022). Spin–photon coupling for atomic qubit devices in silicon. Phys. Rev. Appl..

[31] Fredkin E, Toffoli T (1982). Conservative logic. Int. J. Theor. Phys..

[32] Waldherr G (2014). Quantum error correction in a solid-state hybrid spin register. Nature.

[33] Foletti S, Bluhm H, Mahalu D, Umansky V, Yacoby A (2009). Universal quantum control of two-electron spin quantum bits using dynamic nuclear polarization. Nat. Phys..

[34] Morello A (2010). Single-shot readout of an electron spin in silicon. Nature.

[35] Keith D (2022). Ramped measurement technique for robust high-fidelity spin qubit readout. Sci. Adv..

[36] Hsueh Y-L (2023). Hyperfine-mediated spin relaxation in donor-atom qubits in silicon. Phys. Rev. Res..

[37] Dehollain JP (2012). Nanoscale broadband transmission lines for spin qubit control. Nanotechnology.

[38] Philips SGJ (2022). Universal control of a six-qubit quantum processor in silicon. Nature.

[39] Mills AR (2022). Two-qubit silicon quantum processor with operation fidelity exceeding 99%. Sci. Adv..

[40] Veldhorst M (2014). An addressable quantum dot qubit with fault-tolerant control-fidelity. Nat. Nanotechnol..

[41] Cerezo M (2021). Variational quantum algorithms. Nat. Rev. Phys..

[42] Bharti K (2022). Noisy intermediate-scale quantum algorithms. Rev. Mod. Phys..

[43] Fuechsle M (2012). A single-atom transistor. Nat. Nanotechnol..

[44] Rabeau JR (2006). Implantation of labelled single nitrogen vacancy centers in diamond using N15. Appl. Phys. Lett..

[45] Togan E (2010). Quantum entanglement between an optical photon and a solid-state spin qubit. Nature.

