# Supplementary material for: High-fidelity initialization and control of electron and nuclear spins in a four-qubit register
Source: Nat Nanotechnol. 2024 Feb 7;19(5):605–11. doi: 10.1038/s41565-023-01596-9 (PMC11106007; doi:10.1038/s41565-023-01596-9)
Supplement: Supplementary file 1 — Supplementary discussion with six sections, four figures and three tables. [file 41565_2023_1596_MOESM1_ESM.pdf]

nature nanotechnology

Article https://doi.org/10.1038/s41565-023-01596-9

High-fidelity initialization and control of electron and nuclear spins in a four-qubit register

In the format provided by the authors and unedited

CONTENTS

I. NEMO-3D modelling of donor position 2

II. Determining the EDSR resonance frequencies 2

III. Optimisation of adiabatic inversion pulses for EDSR 3

IV. Randomise and measureme procedures for determining the intiailisation fidelity 5

V. Nuclear spin initialisation fidelitiy calculations and $|\Uparrow\Uparrow\Uparrow\rangle$ initialisation 6

VI. Randomised Benchmarking parameters 8

References 9

I. NEMO-3D MODELLING OF DONOR POSITION

The qubit device was patterned on a 45 nm isotopically purified silicon-28 (∼200 ppm of residual spinful isotopes) buffer layer that decouples the device from the nuclear spin bath of the natural silicon substrate. After dosing with phosphine gas and an incorporation anneal at 350°C for 60 s the devices are encapsulated in 45 nm of ^{28}Si at 250°C and at a rate of 0.15 nm/min to ensure high-quality epitaxy and to limit the thermal budget to avoid donor segregation and diffusion.

Scanning tunneling microscopy hydrogen lithography currently offers an unprecedented accuracy in the placement of single P dopant atoms as qubits with ± 1 atomic lattice site accuracy [1]. This overall placement accuracy is maintained for the position of the P dopants within a small quantum dot (< 1.5 nm^2), such as the ones that form our multi-nuclear spin registers. However, the relative position and number of the P atoms that incorporate within this dot are determined by the stochastic nature of the phosphine incorporation process and the exact size of the patch. Here the small phosphine (PH_3) molecule has to lose all its' hydrogen atoms before the phosphorus atoms can incorporate into the lattice. When the PH_3 gas absorbs to the silicon surface it immediately loses one its hydrogen atoms to form PH_2 + H across one dimer within the lithographic patch. The process of losing the remaining two hydrogen atoms occurs with PH_x species on adjacent dimers, hence the ±1 atomic lattice site accuracy. Depending on the PH_x species present on neighbouring silicon dimers we have an uncertainty of ±1P in the total number of dopants that incorporate in such small patch sizes due to the stochastic nature of the chemical reaction process. Thus, we confirm that the final location of the phosphorus atoms and the number within the quantum dot are again known with unprecedented accuracy (±1 atomic lattice site and ±1P accuracy respectively). Several strategies are currently being pursued globally to achieve *absolute* lattice precision dopant accuracy, both in the exact number of dopants and their precise location:

- elevated temperature (150°C) dosing [2, 3],
- low temperature (77K) dosing [4] with initial results promising,
- tip assisted incorporation, in which absolute lattice precision has already been successfully demonstrated [5].

In our paper, we have not yet employed these new techniques, but they remain active areas of investigation.

The exact arrangement of the phosphorus donor atoms within the multi-nuclear spin register in the silicon lattice has a significant impact on the operation of the spin qubit. The presence of 3P atoms in the register is confirmed by the observation of the 8-fold ESR spectrum. From the ESR spectra we can directly determine the hyperfine couplings: $A_{L,1} = 6 \pm 0.5$ MHz, $A_{L,2} = 68 \pm 0.5$ MHz and $A_{L,3} = 101 \pm 0.5$ MHz for the left quantum dot, and $A_{R,1} = 42 \pm 1.0$ MHz, $A_{R,2} = 76.0 \pm 1.0$ MHz and $A_{R,3} = 200 \pm 1.0$ MHz for the right quantum dot. These distinct hyperfine coupling values can be used to determine the exact atomic configuration of the phosphorus atoms within the registers based on the semi-empirical tight-binding modeling tool NEMO-3D [6, 7]. This framework uses a $sp^3d^5s^*$ atomic orbital and spin basis to express the electronic wavefunction inside the Si crystal. Each phosphorus donor is modelled as a screened Coulomb potential with a central-cell correction. Hyperfine calculations are performed by evaluating the electron densities at the donor sites. The model has been used before to successfully determine the donor positions within the phosphorus multi-donor quantum dots using their hyperfine coupling values [8, 9]. Besides hyperfine based donor metrology, this methodology has also demonstrated good agreement with other experimental results from the Si:P system such as multi-electron charging energies [10] and spin relaxation times [11].

TABLE SI. Comparison between hyperfine coupling values obtained from experiments and NEMO-3D simulations of the donor configurations from Fig. 1(d) of the main text.

		Experiment	NEMO-3D
L - 3P	$A_{L,1}$ (MHz)	6±0.5	7.6
	$A_{L,2}$ (MHz)	68±0.5	65.9
	$A_{L,3}$ (MHz)	101±0.5	102.5
R - 3P	$A_{R,1}$ (MHz)	42±1.0	43.2
	$A_{R,2}$ (MHz)	76±1.0	77
	$A_{R,3}$ (MHz)	200±1.0	196.3

To determine the likely donor positions within each register, we calculate the hyperfine couplings for all possible in-plane and out-of-plane 3P configurations of the donors within a region in the Si crystal that is slightly larger (~ 0.764 nm) than the STM lithographic patch of the corresponding register to account for diffusion. For the donor positions shown inside both the left and right registers in Fig. 1(d) of the main text, we obtain good agreement between the hyperfine values obtained in the experiment and NEMO-3D simulations as shown in Table SI.

II. DETERMINING THE EDSR RESONANCE FREQUENCIES

The EDSR frequencies correspond to flip-flop transitions where the electron spin swaps its spin state with the nuclear spin, such that the total angular momentum is conserved. To determine the values of these frequencies we find the energies of the spin states from the Hamiltonian in Equation 1 of the main text. To first order, the resonance frequency corresponding to the EDSR transition $|\downarrow \cdots \Uparrow_i \cdots\rangle \longleftrightarrow |\uparrow \cdots \Downarrow_i \cdots\rangle$ where the nuclear

TABLE SII. ESR and EDSR resonance frequencies for the 3P spin register with $A_1 = 6$ MHz, $A_2 = 68$ MHz, and $A_3 = 101$ MHz at $B_0 = 1.447$ T.

Transition	Theo. EDSR (GHz)	Exp. EDSR (GHz)
1	40.4861	40.4859 ± 0.0005
2	40.5164	40.5162 ± 0.0005
3	40.5225	40.5222 ± 0.0005
4	40.5345	40.5345 ± 0.0005
5	40.5400	40.5405 ± 0.0005
6	40.5526	40.5526 ± 0.0005
7	40.5891	40.5889 ± 0.0005
8	40.6013	40.6013 ± 0.0005
9	40.6074	40.6074 ± 0.0005
10	40.6197	40.6197 ± 0.0005
11	40.6256	40.6256 ± 0.0005
12	40.6559	40.6556 ± 0.0005

spin i flip-flops with the electron spin is given by,

$$f_{EDSR} = \gamma_e B + |\gamma_n| B + \frac{1}{2} \sum_{j \neq i} \langle n_j | \, \sigma_{zj} \, | n_j \rangle \, A_j \qquad (1)$$

Here, $|n_j\rangle$ is the state of the nuclear spin j and σ_{zj} is the Pauli matrix σ_z operating on this nuclear spin. The matrix element $\langle n_j | \, \sigma_{zj} \, | n_j \rangle$ calculates the spin z-projection of the nuclear spin j, therefore, it is $+1(-1)$ when the spin state is $|\Uparrow\rangle$ $(|\Downarrow\rangle)$. We use this equation to determine all the EDSR resonance frequencies in the main text.

In Table SII we show the EDSR transition frequencies for the 3P multi-nuclear spin register in the main text. The calculated values are in perfect agreement with the experiment within the measurement error, determined by the frequency sampling resolution of 0.25 MHz.

The magnetic field strength of 1.45T used in the main text was chosen to maximise the electron spin readout fidelity by making the qubit frequency (Zeeman energy) larger than the thermal energy of the electron reservoir, while minimizing the degradation of electron spin relaxation time at large magnetic fields [12]. Additionally, at 1.5T the qubit frequency is already approximately 42 GHz, a frequency which scales linearly with magnetic field. Going to higher magnetic fields is challenging due to the difficulty in engineering good signal transmission at these high frequencies.

III. OPTIMISATION OF ADIABATIC INVERSION PULSES FOR EDSR

As mentioned in the main text, the electric fields used to drive the EDSR transitions of the multi-nuclear spin registers were generated from the on-chip broadband antenna fabricated above the phosphorus device layer [8, 13]. The antenna was designed for broadband transmission to generate magnetic fields from a few MHz up to tens of GHz. This was achieved by optimizing the antenna geometry from a coplanar waveguide structure down to a stripe-line (single short) where the magnetic field is maximal. The antenna is positioned such that the multi-nuclear spin registers were located within the antenna loop as shown in [8]. The multi-nuclear registers are estimated to be ~450 nm from the short of the antenna (which is ~725 nm long) and offset by ~90 nm from the centre of the loop. The antenna was used to drive both the magnetic and electric transitions in the register. Despite being optimized for magnetic control of the qubit, we also obtained large enough electric fields from our antenna at ~39 GHz that were used to perform EDSR. In future devices, we aim to optimize the antenna and/or electrostatics gates to improve the speed of electrical control to increase the Rabi frequency of the EDSR transitions.

To characterise the efficiency of the adiabatic inversion pulses we perform Landau-Zener measurements on 3 of the EDSR transitions (one transition for each nuclear spin). First, we initialise the system into the $|\uparrow\Uparrow\Uparrow\Uparrow\rangle$ state (similar to the $|\downarrow\Downarrow\Downarrow\Downarrow\rangle$ described in the main text, but with the electron spin inverted to the $\uparrow$ state using ESR between EDSR pulses). We then perform an adiabatic inversion pulse at the target EDSR transition (9, 11, or 12 flipping each nuclear spin separately), in which the frequency is swept linearly around the transition frequency (chirp), as detailed below. The inversion rate strongly depends on the rate of the frequency sweep w/τ (τ is the pulse duration, w is the frequency width of the sweep) compared to the driving strength f_r (here expressed in terms of the corresponding Rabi frequency), as shown by the Landau-Zener formula:

$$P = 1 - \exp\left(-\frac{\pi^2 f_r^2 \tau}{w}\right) \qquad (2)$$

To determine the optimal ramp rate for efficient adiabatic inversion we vary the pulse duration τ while keeping the chirp width fixed at $w = 1$ MHz and measure the electron spin inversion probability. Fig. S1a shows this inversion probability for EDSR transitions 9, 11 and 12, each corresponding to a nuclear spin flip of n_3, n_2 and n_1, respectively, when starting from the $|\downarrow\Uparrow\Uparrow\Uparrow\rangle$ state. Fitting the measured inversion rate with Eq. 2, we find Rabi frequencies $f_{r,1} = 11.12 \pm 0.42$ kHz, $f_{r,2} = 23.78 \pm 1.48$ kHz and $f_{r,3} = 6.47 \pm 0.38$ kHz at a power of 0 dBm at the microwave vector signal generator output. By varying the microwave power we confirm the expected linear dependence of the driving frequency on the square-root power, but with different slopes for each nuclear spin, n_1, n_2, and n_3 (Fig. S1b). To explain this observation we assume that the microwave-induced $\mathbf{E}$ is equal at all 3 transition frequencies and donor locations. The different slopes therefore represent different electric dipolar moments of the individual donors, coupling them to the electric drive with different strengths. As we will now show, to first order the coupling strength is given by the Stark shift created by the ac electric field amplitude

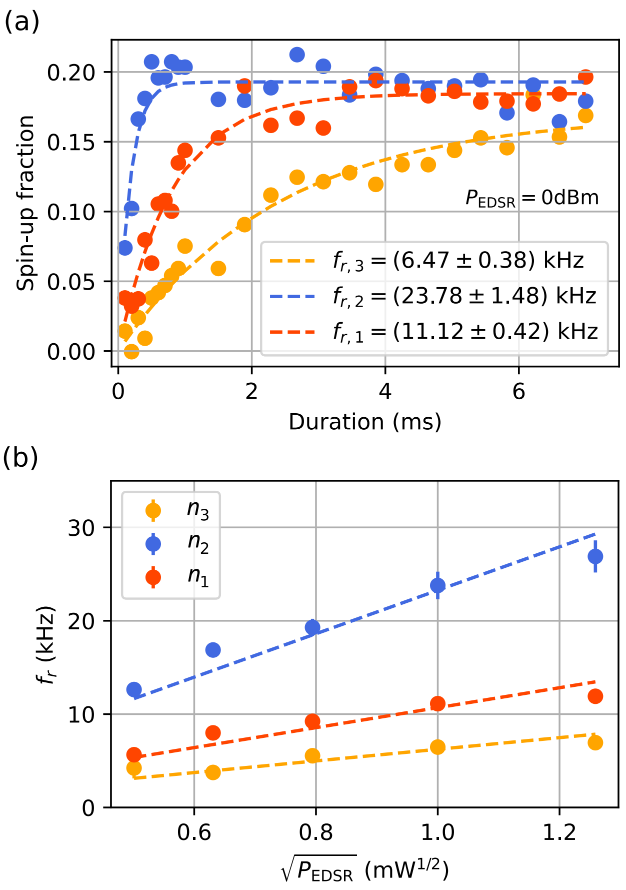

FIG. S1. **Optimisation of the adiabatic inversion for EDSR initialisation.** a) Landau-Zener EDSR transitions for each nuclear spin. The adiabatic inversion pulse duration is varied with the resulting electron spin-up probability showing the efficiency of the inversion pulse. This probability plateaus for longer pulses corresponding to full inversion of the electron spin. b) The experimentally determined Rabi frequency, f_r as a function of EDSR power for each nuclear spin obtained from fitting the spin-up probability in a). Each nuclear spin has a different Rabi frequency dependence on EDSR power due to Stark shift variation in the register.

$\delta A_i = \eta_i \epsilon$.

From tight-binding simulations, we derive the Rabi frequencies of the EDSR drive for individual nuclear spins. Remarkably, we can also obtain an estimation of the ac electric field radiated from the antenna at the dot location, as we discuss below. The hyperfine coupled donor-electron system in question has a static spin Hamiltonian of the form $H_0 = \omega_n I_z + \omega_e S_z + A\mathbf{I}\cdot\mathbf{S}$, where, $\omega_n(\omega_e)$ is the donor nuclear (electron) Larmor frequency, $\mathbf{I}(\mathbf{S})$ is the nuclear (electron) spin operator and A is the Fermi contact hyperfine constant. EDSR causes nuclear-electron spin flip-flop transitions with the relevant $I_z + S_z = 0$ subspace Hamiltonian,

$$H_0 = \begin{pmatrix} -\frac{\omega_e}{2} + \frac{\omega_n}{2} - \frac{A}{4} & \frac{A}{2} \\ \frac{A}{2} & \frac{\omega_e}{2} - \frac{\omega_n}{2} - \frac{A}{4} \end{pmatrix} \quad (3)$$

For systems with >1P the lack of spherical symmetry gives rise to a finite dipole moment and a linear Stark shift. When an ac electric field is applied, it varies the hyperfine coupling and therefore affects the energies of the spin states through the Stark effect. For a small ac electric field, the linear Stark shift is the dominant term (described later in this section), such that we can express the ac electrical drive (in the $I_z + S_z = 0$ subspace) as,

$$H' = \Delta A(t)\mathbf{I}\cdot\mathbf{S} = \eta\epsilon\cos\omega t \begin{pmatrix} -\frac{1}{4} & \frac{1}{2} \\ \frac{1}{2} & -\frac{1}{4} \end{pmatrix} \quad (4)$$

Here, η is the linear Stark coefficient and ϵ is the amplitude of the ac electric field. By transferring to the eigenbasis of H_0, using a Rotating Wave Approximation and choosing a convenient origin for the energy to eliminate offsets, we have,

$$H = H_0 + H' = \begin{pmatrix} \frac{1}{2}(\omega_0 - \omega) & \frac{\eta\epsilon}{4} \\ \frac{\eta\epsilon}{4} & -\frac{1}{2}(\omega_0 - \omega) \end{pmatrix} \quad (5)$$

where, ω_0 is the eigen-splitting of H_0. Here we use $\omega_e \gg \omega_n, A$ to make the approximation $\eta\epsilon\frac{\omega_n - \omega_e}{4\sqrt{A^2 + (\omega_n - \omega_e)^2}} \approx \frac{\eta\epsilon}{4}$. Hence the Rabi frequency of the drive is $\frac{\eta\epsilon}{2}$ at the resonance frequency.

Due to the close proximity of the donors, the ac electric field is nominally the same at all the donor locations. It means that the Stark coefficients are proportional to the Rabi frequencies with the proportionality constant being half the amplitude of the ac electric field.

For a small electric field $\boldsymbol{\epsilon}$ applied on top of a dc electric field $\boldsymbol{\epsilon}_0$ the hyperfine constant $A_i(\boldsymbol{\epsilon}_0 + \boldsymbol{\epsilon})$ of donor i can be written up to first order as,

$$\begin{aligned} A_i(\boldsymbol{\epsilon}_0 + \boldsymbol{\epsilon}) &= A_i(\boldsymbol{\epsilon}_0) + \boldsymbol{\epsilon}\cdot\nabla_{\boldsymbol{\epsilon}} A_i|_{\boldsymbol{\epsilon}=\boldsymbol{\epsilon}_0} \\ \Rightarrow \Delta A_i &= \boldsymbol{\epsilon}\cdot\nabla_{\boldsymbol{\epsilon}} A_i|_{\boldsymbol{\epsilon}=\boldsymbol{\epsilon}_0} \end{aligned} \quad (6)$$

Here, ΔA_i is the hyperfine Stark shift of donor i and we define $\nabla_{\boldsymbol{\epsilon}} A_i|_{\boldsymbol{\epsilon}=\boldsymbol{\epsilon}_0} = \boldsymbol{\eta}_i$ as the Stark coefficient vector for donor i. It tells us that the electric field response of the electron density at a donor site depends on the direction of the field. For a field along $\hat{\mathbf{n}}$, the Stark coefficient is $\eta_i = \boldsymbol{\eta}_i \cdot \hat{\mathbf{n}}$, used in Eqn. 5.

We now calculate the linear Stark coefficient vectors ($\boldsymbol{\eta}_i$) for the donors in this particular configuration using NEMO-3D. Since the register has a planar configuration, the electric dipole is only significant in the xy plane; hence, only the x and y components of the electric field are considered. We apply an electric field of 0.5 MV/m separately along the x and y directions to perform two simulations to confirm the hyperfine values. We confirm that the Stark shift is primarily linear up to electric fields

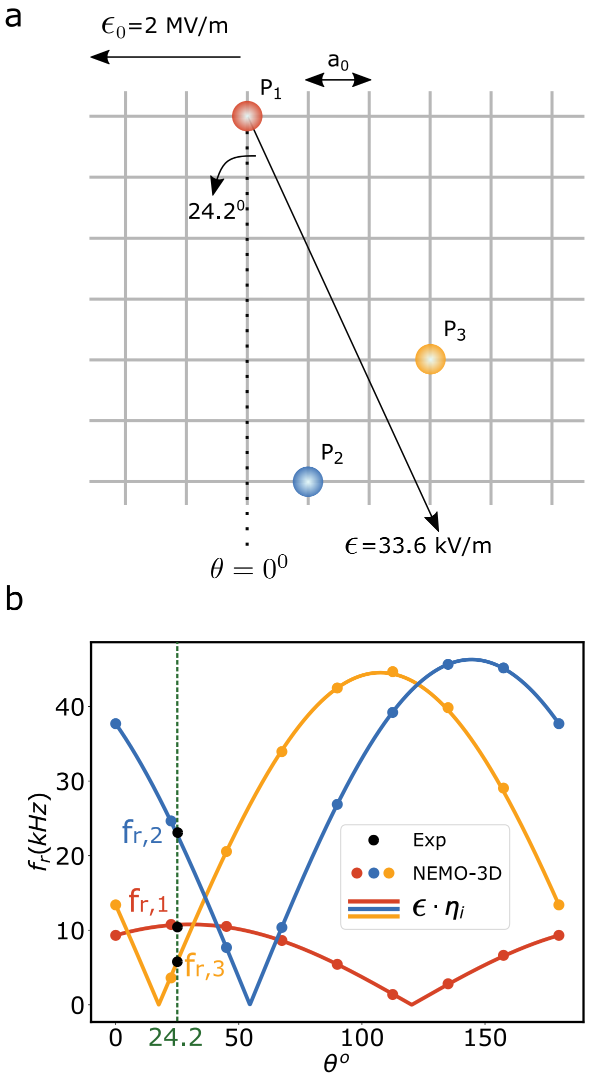

FIG. S2. **Determination of the ac Stark coefficients from tight-binding simulations.** a) Amplitude and direction of the ac electric field applied to the 3P register. b) Variation of the Rabi frequencies, f_r with the direction of the ac electric field polarization when the electric field magnitude is fixed at 33.6 kV/m. At an angle of 24.2^0 the Rabi frequencies $f_{r,1}, f_{r,2}$ and $f_{r,3}$ agree with experimental results as shown in Table SIII

of the order of ∽1 MV/m. From the electric field simulations along x and y we obtain the x and y components of $\boldsymbol{\eta}_i$ for each donor.

The values of $\boldsymbol{\eta}_i$, combined with the measured $f_{r,\ i}$, constrain the direction and the amplitude of the ac electric field at the qubit position and allow us to extract both of them. From the values of $\boldsymbol{\eta}_i$ we calculate $\boldsymbol{\eta}_i \cdot \hat{\mathbf{n}}$ by varying the direction of the unit vector $\hat{\mathbf{n}}$ and look for the direction where the values of $\boldsymbol{\eta}_i \cdot \hat{\mathbf{n}}$ are proportional to the $f_{r,\ i}$'s obtained from experiments. We find that $\epsilon = 33.6 \pm 0.5$ kV/m polarized at an angle 24.2° defined in Fig. S2(a) gives Rabi frequencies in excellent

TABLE SIII. Comparison of Rabi frequencies between experiments at 0 dBm and NEMO-3D simulations

	$f_{r,1}$ (kHz)	$f_{r,2}$ (kHz)	$f_{r,3}$ (kHz)
Experiment	11.12 ± 0.42	23.78 ± 1.48	6.47 ± 0.38
NEMO-3D	11.30 ± 0.17	23.81 ± 0.35	5.92 ± 0.09

agreement with the 0 dBm measurements shown in Table SIII. Fig. S2(b) shows the variation of $f_{r,\ i}$ of each donor within the register with the direction of this ac electric field. The ac electric field value agrees with electric fields simulated using finite-element modelling of the antenna [13]. Importantly, future re-designs of the device architecture can allow the microwave pulse to be applied to the in-plane gate electrodes, which would induce a stronger and more local field, allowing faster coherent operation. Finally, from the Landau-Zener calibration curve (Fig. S1a) we choose an optimal pulse duration, trading-off between device heating, and the overhead associated with longer initialisation pulse durations at lower driving powers. At 0 dBm, the inversion rate of I_2 and I_3 approaches unity for $\tau > 4$ ms, but is only $\sim 70\%$ for I_1, which requires $\tau > 10$ ms for full inversion due to the low Rabi frequency of the EDSR transitions. As a compromise we choose $\tau = 5$ ms for the actual initialisation sequence, to avoid making the sequence duration prohibitively long compared to the circuit execution time.

IV. RANDOMISE AND MEASUREME PROCEDURES FOR DETERMINING THE INTAILISATION FIDELITY

In this section we detail the additional randomise and measure steps for quantifying the intialisation procedure described in the main text.

Randomise: The initialisation fidelity will ultimately depend on the initial state of the system since we need to flip up to 3 nuclear spins. To account for this variability we perform a randomisation step to access all the possible initial nuclear spin states to determine an average initialisation fidelity. We first load a random electron spin state by pulsing quickly across the $0 \rightarrow 1$ charge transition line of the left quantum dot [14]. We then perform an electron-nuclear SWAP gate by applying an adiabatic inversion pulse at the 4 EDSR transition frequencies for the same nuclear spin (Fig **??**c), thereby driving this nuclear spin to a random initial state. We then apply the same sequence for the other two nuclear spins (EDSR 1, 6, 7, and 12 for n_1, EDSR 2, 3, 10, and 11 for n_2, and EDSR 4, 5, 8, and 9 for n_3), and repeat 50 times to ensure that all the nuclear spins are fully mixed. To determine the starting state of the register we then subsequently measure the state of each nuclear spin after randomisation, described in the next step.

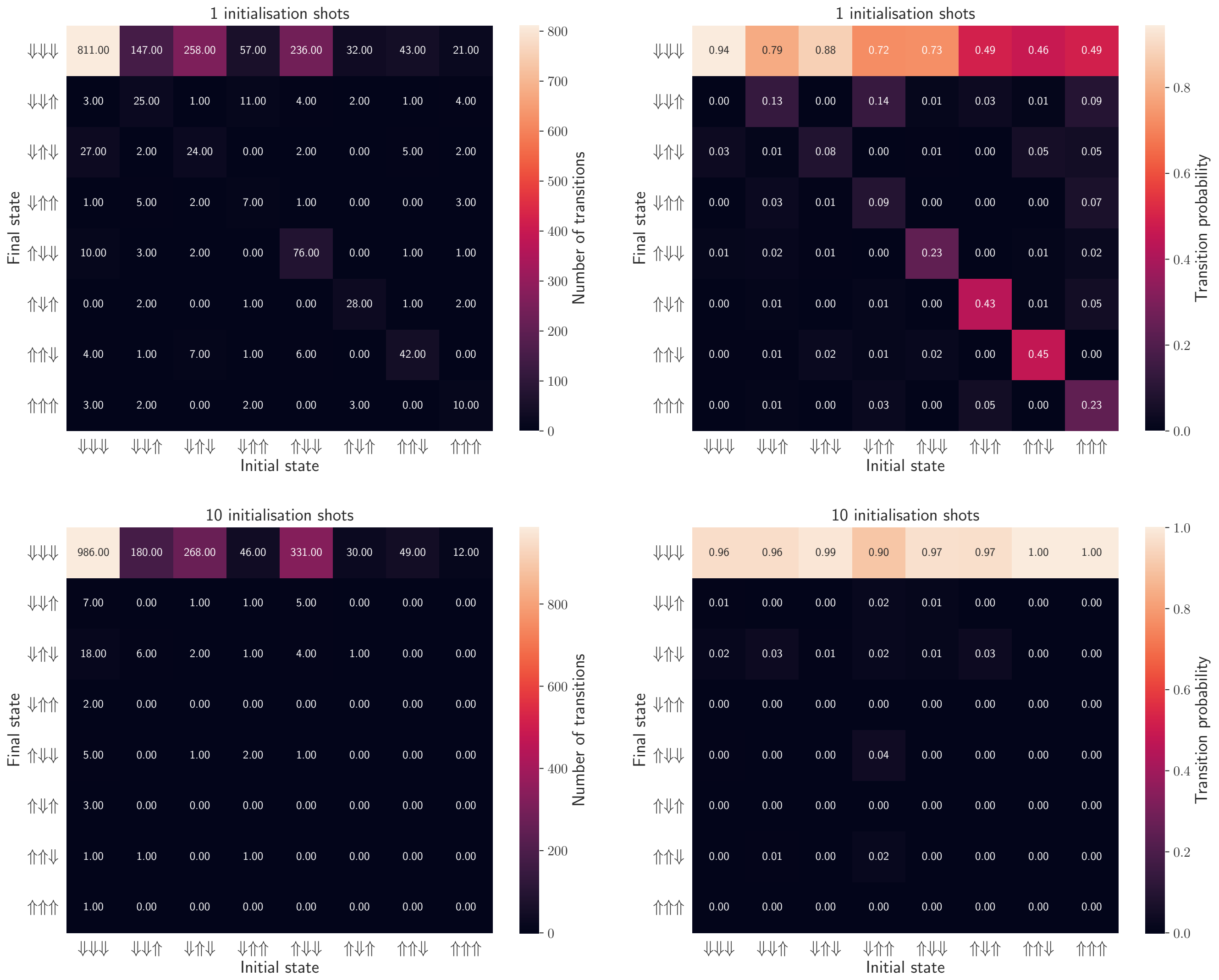

FIG. S3. **Experimental transition matrices used to determine the nuclear spin initialisation fidelity.** Top left: The raw incident count matrix, $M_{j,i}$ for a single initialisation shot. Each element in the matrix corresponds to number of instances starting in state i and measuring state j after initialisation. Top right: The raw count matrix is then converted to a probability matrix, $P_{j,i}$ to determine the probability of correctly initialising the nuclear spin register into the $|\Downarrow\Downarrow\Downarrow\rangle$ state. Bottom row: The same matrices as the top row but for 10 initialisation shots. Here, we can see the initialisation fidelity (top row in the matrix approaches unity) is significantly improved.

Measure: The measurement of the nuclear spin states involves first initialising the electron spin state into $|\downarrow\rangle$. Then, we perform an electron spin inversion at the ESR frequency of the first nuclear spin resonance $|\Downarrow\Downarrow\Downarrow\rangle$ and then measure whether the electron spin has flipped via energy-selective readout, see Fig. **??**a. This measurement constitutes a nuclear spin state readout of $|\Downarrow\Downarrow\Downarrow\rangle$, that is, if the electron spin was measured to be $|\uparrow\rangle$ then we know the nuclear spins were in the $|\Downarrow\Downarrow\Downarrow\rangle$ state. This measurement procedure (initialise electron spin, ESR inversion, and electron spin measurement) is performed for each of the 8 different ESR transition frequencies and averaged 60 times to determine the overall starting nuclear spin state with high fidelity. This starting state is then used for the nuclear spin initialisation fidelity characterization.

V. NUCLEAR SPIN INITIALISATION FIDELITIY CALCULATIONS AND $|\Uparrow\Uparrow\Uparrow\rangle$ INITIALISATION

The fidelity of initialising the nuclear spins into the $|\Downarrow\Downarrow\Downarrow\rangle$ or $|\Uparrow\Uparrow\Uparrow\rangle$ state is obtained from the following measurement sequence (described in the main text and Fig. 4(a). First, we **randomise** the nuclear spins and repeat this 5 times. Then we perform a **measurement** of

the nuclear spin state. Due to the relatively low electron spin readout fidelity in this particular device ($\sim 81\%$), it is necessary to repeat the entire readout sequence 60 times to determine the nuclear spin state with fidelity $> 99\%$. Afterwards, we **initialise** the nuclear spins into the $|\Downarrow\Downarrow\Downarrow\rangle$ or $|\Uparrow\Uparrow\Uparrow\rangle$ state, with a variable number of initialisation shots. Finally, another nuclear spin readout is performed (same parameters as before). In this way, the nuclear spin state is determined before and after the initialization shots. The whole experiment is repeated 2000 times for statistical significance.

In the left column of Fig. S3 we plot the transition matrix, $M_{j,i}$ for the $|\Downarrow\Downarrow\Downarrow\rangle$ initialisation containing the number of transitions from any initial state to all final states, for a single initialisation shot (top row) and 10 shots (bottom row). The corresponding transition probabilities, $P_{j,i}$ shown in the right column of Fig. S3, are calculated by normalising each matrix element with respect to the sum in the corresponding column, requiring that regardless of the initial state, the probability at the end in all of the 8 nuclear spin states should be unity. The fidelity shown in Fig. 4(c) in the main text, is equal $P_{\Downarrow\Downarrow\Downarrow,i}$ for any initial state i except $|\Downarrow\Downarrow\Downarrow\rangle$. We further normalise the fidelity by $\bar{P}_{\Downarrow\Downarrow\Downarrow,\Downarrow\Downarrow\Downarrow}$ the mean value of $P_{\Downarrow\Downarrow\Downarrow,\Downarrow\Downarrow\Downarrow}$ across all experiments. By normalising this value we are making an implicit assumption that nuclear spin preparation and measurement errors are the leading source of error in this analysis. That is, the initialisation fidelity of state i is given by,

$$F_i = P_{\Downarrow\Downarrow\Downarrow,i}/\bar{P}_{\Downarrow\Downarrow\Downarrow,\Downarrow\Downarrow\Downarrow} \quad (7)$$

and the uncertainties calculated using,

$$\sigma_{F,i} = (\sigma_{\Downarrow\Downarrow\Downarrow} + \sqrt{F_i/N_i})/2 \quad (8)$$

where N_i is the number of times the nuclear spins were measured in state i.

Importantly, we can modify the pulse sequence to initialise the nuclear spin register into any state. Since the EDSR transitions swap the electron and nuclear spin states, if we first perform an electron spin inversion before applying the SWAP operations then the nuclear spin will be automatically initialised into $|\Uparrow\rangle$. The corresponding circuit for the alternate nuclear spin initialisation in the $|\Uparrow\Uparrow\Uparrow\rangle$ state is thus given by Fig. S4.

To further investigate the performance of the electron spin qubit as a function of the initialized nuclear spin state, in Fig. S5 we show Rabi and Ramsey oscillation measurements of the electron spin after initialization to each of the 8 different nuclear spin states. We find that the dephasing time measured through the Ramsey oscillations varies across the sample without any trend and is most likely due to variations in the system dynamics, see Tab. SIV. The Rabi frequency also varies with resonance frequency due to small variations in the transmission through the antenna lines in the dilution refrigerator as seen in Ref. [15]. We note that these measurements were taken in a different cooldown on a different dilution refrig-

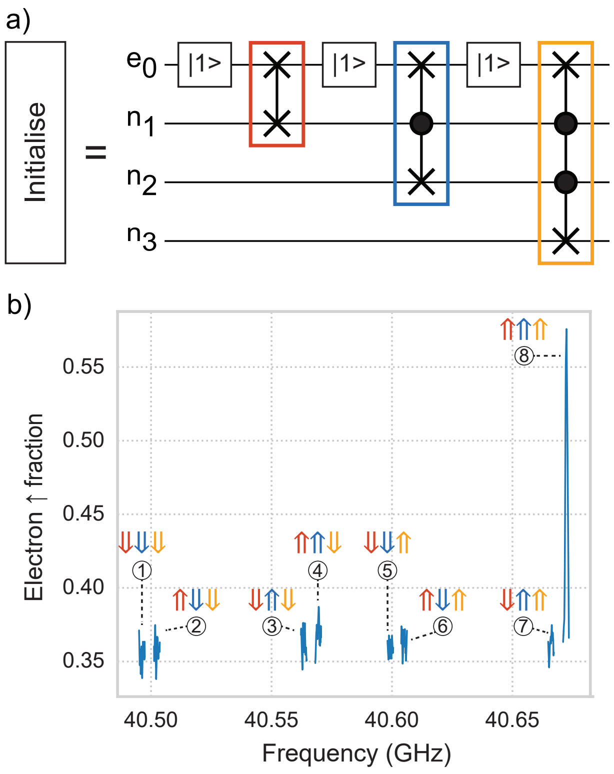

FIG. S4. **Nuclear spin initialisation sequence for $|\Uparrow\Uparrow\Uparrow\rangle$.** a) The same nuclear spin initialisation protocol as described in the main text except for the initialisation of the electron spin the $|1\rangle$ ($|\uparrow\rangle$) state. The electron-nuclear SWAP gate of the EDSR transitions now swaps the electron and nuclear spin state so that the nuclear spins are now initialised in $|\Uparrow\Uparrow\Uparrow\rangle$. b) The resulting ESR spectrum after performing initialisation into the $|\Uparrow\Uparrow\Uparrow\rangle$ showing polarisation into the highest ESR resonance.

TABLE SIV. Summary of the electron spin qubit dephasing and Rabi frequency for different nuclear spin states from a different cool down of the device. The EDSR initialization procedure was used to realise each of the eight different nuclear spin states and then a Rabi and Ramsey measurement were taken.

Nuclear state	Rel. frequency (MHz)	T_2^* (μs)	f_{Rabi} (kHz)
$\lvert\Downarrow\Downarrow\Downarrow\rangle$	0	30	92
$\lvert\Uparrow\Downarrow\Downarrow\rangle$	6	33	94
$\lvert\Downarrow\Uparrow\Downarrow\rangle$	68	35	109
$\lvert\Uparrow\Uparrow\Downarrow\rangle$	74	38	109
$\lvert\Downarrow\Downarrow\Uparrow\rangle$	103	29	106
$\lvert\Uparrow\Downarrow\Uparrow\rangle$	109	33	104
$\lvert\Downarrow\Uparrow\Uparrow\rangle$	171	31	58
$\lvert\Uparrow\Uparrow\Uparrow\rangle$	177	31	61

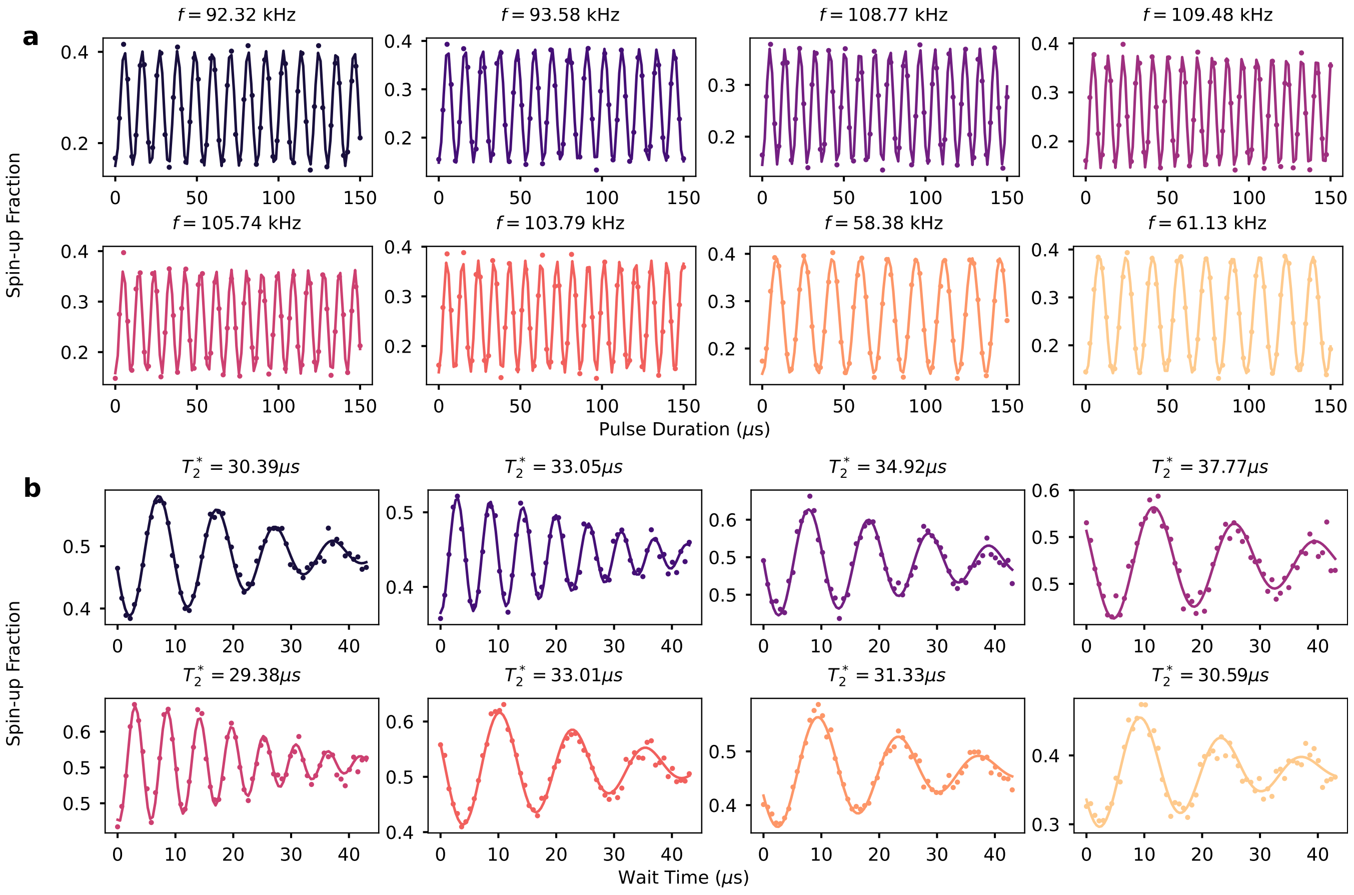

FIG. S5. **Electron spin qubit performance for different initialised nuclear spin states.** a) Rabi oscillation measurements for different nuclear spin configurations ordered from lowest frequency ($|\Downarrow\Downarrow\Downarrow\rangle$) to highest frequency ($|\Uparrow\Uparrow\Uparrow\rangle$). Here, the Rabi frequency varies $\sim 90 \pm 20$ kHz due to the slightly different transmission of the microwave signal down the fridge due to resonances in the dilution refrigerator wiring around 39 GHz. b) Ramsey oscillations of the electron spin for the same nuclear spin configurations in a). The coherence time of the electron spin does not vary considerably for the different nuclear spin configurations and is 32 ± 3 μs.

erator. These measurements had been significantly optimized with a different measurement setup (AWG, control voltages, DAQ, and wiring were all changed) meaning that the coherence times and Rabi frequencies quoted here cannot be directly compared to the coherence times in the main text. The improvement to the coherence time is most likely related to the reduced measurement time of the Rabi and Ramsey oscillations compared to the main text [16].

VI. RANDOMISED BENCHMARKING PARAMETERS

Randomised benchmarking [17, 18] is a well-known technique for estimating the fidelity of quantum gates. The protocol consists of applying a sequence of random Clifford gates, followed by a final Clifford gate, chosen randomly from those which ideally recover the qubit to its initial state. However in practice, as the length of the sequence increases, the probability of measuring the qubit in its initial state decreases due to cumulative gate errors. This rate can be fitted as a function of sequence length to estimate the average gate fidelity [19, 20].

The measurement described in the main text uses this protocol, with a variation which recovers that qubit to the $|\uparrow\rangle$-state in addition to the initial $|\downarrow\rangle$-state after each sequence, based on Ref. [21]. By combining these respective measurements, we are able to set one of the fit parameters, namely the spin probability of the fully decayed state, to 50%. This reduces the uncertainty in the fidelity estimate while also eliminating state-preparation and measurement errors.

The protocol was implemented as follows:

1. For sequence lengths $n \in \{2, 5, 13, 36, 97, 256\}$,
   (a) Generate a random sequence of single-qubit Clifford gates $G_n G_{n-1} ... G_1$.
   (b) Classically compute the resulting qubit state $|\psi\rangle = G_n G_{n-1} ... G_1 |\downarrow\rangle$.

(c) Randomly select two recovery gates $R_\uparrow$ and $R_\downarrow$ from the Clifford gates such that $R_\uparrow|\psi\rangle = |\uparrow\rangle$ and $R_\downarrow|\psi\rangle = |\downarrow\rangle$.

(d) Apply the circuits $R_\uparrow G_n G_{n-1}...G_1$ and $R_\downarrow G_n G_{n-1}...G_1$ experimentally using the composition of π- and $\pi/2$-rotation pulses mapped out in [22].

(e) Repeat both circuits 16,000 times and average their spin measurements to produce spin-up probabilities $P_\uparrow^{|\uparrow\rangle}(n)$ and $P_\uparrow^{|\downarrow\rangle}(n)$ respectively.

2. Repeat Step 1. with $k = 10$ random variations and average them for each sequence length n.
3. Combine the $|\uparrow\rangle$- and $|\downarrow\rangle$-recovered distributions using the formula

$$P_\uparrow(n) = \frac{P_\uparrow^{|\uparrow\rangle}(n) + (1 - P_\uparrow^{|\downarrow\rangle}(n))}{2}. \quad (9)$$

4. Estimate the single-qubit Clifford gate fidelity F_C by fitting $P_\uparrow$ with the expression

$$P_\uparrow(n) = P_0(2F_C - 1)^n + P_\infty, \quad (10)$$

where P_∞ is fixed to 50%.

5. Evaluate the single-qubit physical gate fidelity,

$$F_p = 1 - \frac{1 - F_c}{L}, \quad (11)$$

where $L = 1.875$ is the average number of physical gates per Clifford gate.

Combining this procedure with the initialisation protocols in the main text, we were able to measure a single-qubit Clifford gate fidelity of $99.58 \pm 0.14\%$, which corresponds to a single-qubit physical gate fidelity of $99.78 \pm 0.07\%$.

[1] M. Fuechsle, J. A. Miwa, S. Mahapatra, H. Ryu, S. Lee, O. Warschkow, L. C. L. Hollenberg, G. Klimeck, and M. Y. Simmons, A single-atom transistor, Nature Nanotechnology **7**, 242 (2012).

[2] J. A. Ivie, Q. Campbell, J. C. Koepke, M. I. Brickson, P. A. Schultz, R. P. Muller, A. M. Mounce, D. R. Ward, M. S. Carroll, E. Bussmann, A. D. Baczewski, and S. Misra, Impact of incorporation kinetics on device fabrication with atomic precision, Phys. Rev. Appl. **16**, 054037 (2021).

[3] Q. T. Campbell, J. C. Koepke, J. A. Ivie, A. M. Mounce, D. R. Ward, M. S. Carroll, S. Misra, A. D. Baczewski, and E. Bussmann, Quantifying the variation in the number of donors in quantum dots created using atomic precision advanced manufacturing, J. Phys. Chem. C **127**, 6071 (2023).

[4] M. Y. Simmons and J. G. Keizer, A method for selective incorporation of dopant atoms in a semiconductive surface, patent no. united states – 11227768 (2022).

[5] J. Wyrick, X. Wang, P. Namboodiri, R. V. Kashid, F. Fei, J. Fox, and R. Silver, Enhanced atomic precision fabrication by adsorption of phosphine into engineered dangling bonds on H-Si using STM and DFT, ACS Nano **16**, 19114 (2022).

[6] G. Klimeck, S. S. Ahmed, Hansang Bae, N. Kharche, S. Clark, B. Haley, Sunhee Lee, M. Naumov, Hoon Ryu, F. Saied, M. Prada, M. Korkusinski, T. B. Boykin, and R. Rahman, Atomistic simulation of realistically sized nanodevices using nemo 3-d—part i: Models and benchmarks, IEEE Transactions on Electron Devices **54**, 2079 (2007).

[7] G. Klimeck, S. S. Ahmed, N. Kharche, M. Korkusinski, M. Usman, M. Prada, and T. B. Boykin, Atomistic simulation of realistically sized nanodevices using nemo 3-d—part ii: Applications, IEEE Transactions on Electron Devices **54**, 2090 (2007).

[8] S. J. Hile, L. Fricke, M. G. House, E. Peretz, C. Y. Chen, Y. Wang, M. Broome, S. K. Gorman, J. G. Keizer, R. Rahman, and M. Y. Simmons, Addressable electron spin resonance using donors and donor molecules in silicon, Science Advances **4**, eaaq1459 (2018).

[9] L. Kranz, S. K. Gorman, B. Thorgrimsson, S. Monir, Y. He, D. Keith, K. Charde, J. G. Keizer, R. Rahman, and M. Y. Simmons, The use of exchange coupled atom qubits as atomic-scale magnetic field sensors, Advanced Materials **35**, 2201625 (2022).

[10] B. Weber, Y. H. M. Tan, S. Mahapatra, T. F. Watson, H. Ryu, R. Rahman, L. C. L. Hollenberg, G. Klimeck, and M. Y. Simmons, Spin blockade and exchange in coulomb-confined silicon double quantum dots, Nature Nanotechnology **9**, 430 (2014).

[11] Y. L. Hsueh, H. Büch, Y. Tan, Y. Wang, L. C. Hollenberg, G. Klimeck, M. Y. Simmons, and R. Rahman, Spin-lattice relaxation times of single donors and donor clusters in silicon, Physical Review Letters **113**, 246406 (2014).

[12] D. Keith, S. K. Gorman, L. Kranz, Y. He, J. G. Keizer, M. A. Broome, and M. Y. Simmons, Benchmarking high fidelity single-shot readout of semiconductor qubits, New Journal of Physics **21**, 063011 (2019).

[13] J. P. Dehollain, J. J. Pla, E. Siew, K. Y. Tan, A. S. Dzurak, and A. Morello, Nanoscale broadband transmission lines for spin qubit control, Nanotechnology **24**, 015202 (2012).

[14] D. Keith, S. K. Gorman, Y. He, L. Kranz, and M. Y. Simmons, Impact of charge noise on electron exchange interactions in semiconductors, npj Quantum Information **8**, 17 (2022).

[15] M. T. Madzik, S. Asaad, A. Youssry, B. Joecker, K. M. Rudinger, E. Nielsen, K. C. Young, T. J. Proctor, A. D. Baczewski, A. Laucht, V. Schmitt, F. E. Hudson, K. M. Itoh, A. M. Jakob, B. C. Johnson, D. N. Jamieson, A. S. Dzurak, C. Ferrie, R. Blume-Kohout, and A. Morello, Precision tomography of a three-qubit donor quantum processor in silicon, Nature **601**, 348 (2022).

[16] L. Kranz, S. K. Gorman, B. Thorgrimsson, Y. He, D. Keith, J. G. Keizer, and M. Y. Simmons, Exploiting a single-crystal environment to minimize the charge noise on qubits in silicon, Advanced Materials **32**, 2003361 (2020).

[17] J. Emerson, R. Alicki, and K. Życzkowski, Scalable noise estimation with random unitary operators, Journal of Optics B: Quantum and Semiclassical Optics **7**, S347 (2005).

[18] E. Knill, D. Leibfried, R. Reichle, J. Britton, R. B. Blakestad, J. D. Jost, C. Langer, R. Ozeri, S. Seidelin, and D. J. Wineland, Randomized benchmarking of quantum gates, Physical Review A **77**, 012307 (2008).

[19] E. Magesan, J. M. Gambetta, and J. Emerson, Scalable and robust randomized benchmarking of quantum processes, Physical review letters **106**, 180504 (2011).

[20] E. Magesan, J. M. Gambetta, and J. Emerson, Characterizing quantum gates via randomized benchmarking, Physical Review A **85**, 042311 (2012).

[21] J. Muhonen, A. Laucht, S. Simmons, J. Dehollain, R. Kalra, F. Hudson, S. Freer, K. M. Itoh, D. Jamieson, J. McCallum, *et al.*, Quantifying the quantum gate fidelity of single-atom spin qubits in silicon by randomized benchmarking, Journal of Physics: Condensed Matter **27**, 154205 (2015).

[22] J. M. Epstein, A. W. Cross, E. Magesan, and J. M. Gambetta, Investigating the limits of randomized benchmarking protocols, Physical Review A **89**, 062321 (2014).
